# Structural and molecular basis of ZNRF3/RNF43 transmembrane ubiquitin ligase inhibition by the Wnt agonist R-spondin

**DOI:** 10.1038/ncomms3787

**Published:** 2013-11-14

**Authors:** Matthias Zebisch, Yang Xu, Christos Krastev, Bryan T. MacDonald, Maorong Chen, Robert J. C. Gilbert, Xi He, E. Yvonne Jones

**Affiliations:** 1 Division of Structural Biology, Wellcome Trust Centre for Human Genetics, University of Oxford, Oxford OX3 7BN, UK; 2 F.M. Kirby Neurobiology Center, Department of Neurology, Boston Children’s Hospital, Harvard Medical School, Boston, Massachusetts 02115, USA; 3 Key Laboratory for Molecular Enzymology and Engineering of Ministry of Education, College of Life Science, Jilin University, Changchun 130012, China

## Abstract

The four R-spondin (Rspo) proteins are secreted agonists of Wnt signalling in vertebrates, functioning in embryogenesis and adult stem cell biology. Through ubiquitination and degradation of Wnt receptors, the transmembrane E3 ubiquitin ligase ZNRF3 and related RNF43 antagonize Wnt signalling. Rspo ligands have been reported to inhibit the ligase activity through direct interaction with ZNRF3 and RNF43. Here we report multiple crystal structures of the ZNRF3 ectodomain (ZNRF3_ecto_), a signalling-competent Furin1–Furin2 (Fu1–Fu2) fragment of Rspo2 (Rspo2_Fu1–Fu2_), and Rspo2_Fu1–Fu2_ in complex with ZNRF3_ecto_, or RNF43_ecto_. A prominent loop in Fu1 clamps into equivalent grooves in the ZNRF3_ecto_ and RNF43_ecto_ surface. Rspo binding enhances dimerization of ZNRF3_ecto_ but not of RNF43_ecto_. Comparison of the four Rspo proteins, mutants and chimeras in biophysical and cellular assays shows that their signalling potency depends on their ability to recruit ZNRF3 or RNF43 via Fu1 into a complex with LGR receptors, which interact with Rspo via Fu2.

## Abstract (web summary)

R-spondins are secreted factors that potentiate Wnt signalling by inhibiting the degradation of Wnt receptors by transmembrane E3 ubiquitin ligases. Zebisch *et al.* present a panel of crystal structures that reveal how this inhibition occurs.

## Graphical abstract

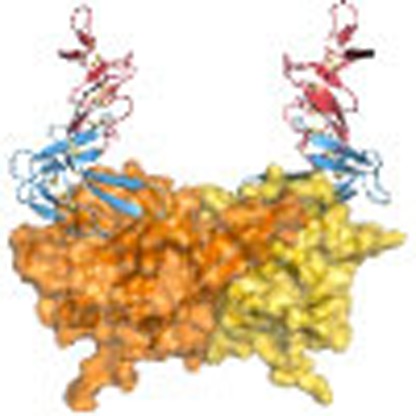

Rspo (R-spondin, also roof plate-specific spondin) proteins are evolutionarily conserved from fish to humans and have well-documented roles in a broad range of developmental and physiological processes resulting from enhancement of canonical and non-canonical Wnt signalling^1^,^2^,^3^. Rspo1 is involved in mammalian sex determination^4^ and is a potent stimulator of epithelial repair in the gastrointestinal tract^2^,^5^. Rspo2 has recently been identified as a major determinant of susceptibility to infectious diarrhoea in mice, linking infection and intestinal homoeostasis^6^. Gene fusions involving *RSPO2* and *RSPO3* have been found in 10% of primary colon cancers^7^, whereas mutations in RSPO4 underlie inherited anonychia, a disorder in nail development^8^,^9^,^10^. The leucine-rich repeat containing G protein-coupled receptors 4, 5 and 6 (LGR4/5/6) are conserved high-affinity cell surface receptors for Rspo proteins^11^,^12^,^13^,^14^,^15^; however, the molecular mechanisms by which Rspo proteins function have remained obscure.

Recently published work has indicated that Rspo proteins can exert their potentiating effects on Wnt signalling through direct interaction with the extracellular regions of ZNRF3 or RNF43, ultimately inducing formation of a complex comprising ZNRF3/RNF43, Rspo and LGR4/5/6 (ref. 16). Similar to the Rspo proteins, ZNRF3 and RNF43 are highly conserved in vertebrates. Loss-of-function mutations of RNF43 in pancreatic cancer have implicated it as a tumour suppressor^17^. ZNRF3 and RNF43 comprise an amino-terminal extracellular region of uncharacterized topology and moderate sequence conservation of 39% identity between the two proteins, a transmembrane region and a cytoplasmic region that bears the hallmark sequence of a really interesting new gene (RING)-type E3 ubiquitin ligase. Similar to LGR4/5/6 receptors, ZNRF3/RNF43 have been reported to associate in the membrane with the Wnt receptor Frizzled and LRP5/6 coreceptors^13^,^16^. ZNRF3/RNF43 specifically targets these Wnt receptors for ubiquitination and turnover, hence reducing Wnt signalling responses^16^,^18^. Direct extracellular interaction with Rspo proteins inhibits ZNRF3/RNF43 activity^16^. These observations have led to the suggestion that Rspo acts to physically bridge between its two receptor types ZNRF3/RNF43 and LGR4/5/6 (ref. 16). Current models suggest that membrane clearance of ZNRF3/RNF43 through this ternary complex relieves turnover of Wnt receptors and hence enhances Wnt responsiveness.

Here we report a molecular level analysis of the ZNRF3/RNF43 ectodomain structure and its interactions with Rspo proteins. Our study provides mechanistic insight into this key control point in the Wnt signalling pathway.

## Results

### Structure determination

**Figure 1:**
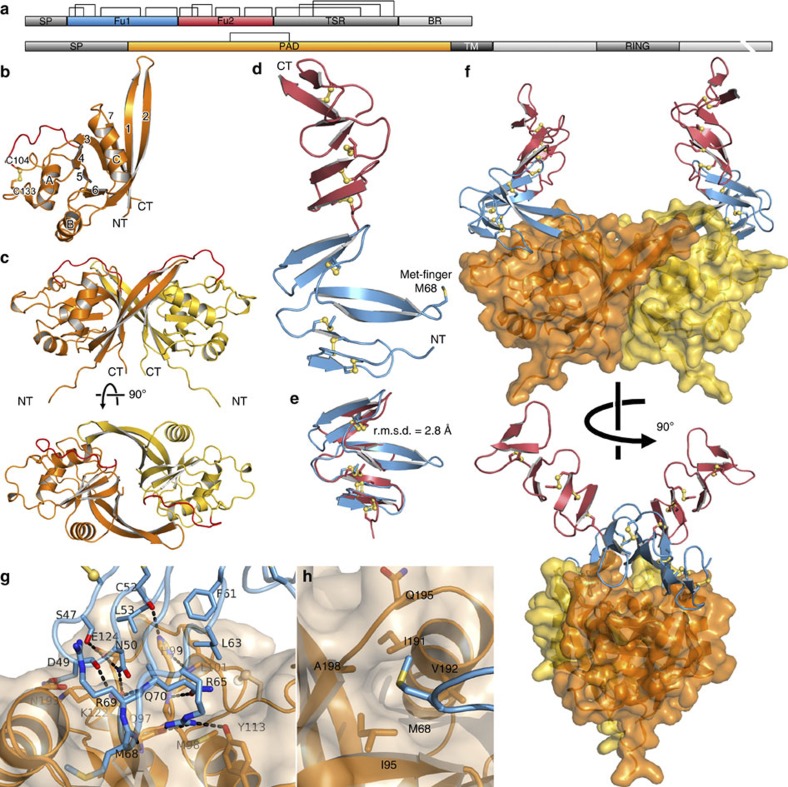
Unliganded and complexed structures of ZNRF3 and Rspo proteins. (**a**) Schematic domain organization of Rspo (top) and ZNRF3/RNF43 proteins (bottom) roughly at scale. The domains included in the crystallization constructs are coloured in blue, red and orange. Disulphides are derived from the crystal structure, except for those of the TSR domain of Rspo, which are based on a model^48^. (**b**) Cartoon representation of the fold of the ZNRF3 ectodomain protomer. β-strands are numbered and α-helices are labelled in alphabetical order from the N to C terminus. (**c**) Structure of the recurring ZNRF3_ecto_ dimer with view parallel to the putative membrane layer and from top towards the membrane. An acidic region with sequence ^105^NNNDEEDLYEY^115^ is highlighted in red in **b** and **c**. (**d**) The xRspo2_Fu1–Fu2_ structure. Both β-hairpins and disulphide bridges line up to form a ladder-like structure. The second β-hairpin of Fu1 contains an exposed methionine side chain. (**e**) Fu1 and Fu2 share the same architecture, except that the second β-hairpin of Fu1 is considerably longer. (**f**) The ZNRF3_ecto_–Rspo2_Fu1–Fu2_ complex as the same 2:2 symmetric complex in all seven crystallographic observations. Shown are two views parallel to the putative membrane orientation. The RNF43_ecto_–Rspo2_Fu1–Fu2_ complex resembles one half of this complex (Supplementary Fig. S5). (**g**) The ZNRF3_ecto_–Rspo2_Fu1–Fu2_ interface. xZNRF3_ecto_ is shown in semi-transparent surface (orange) and ribbon, xRspo2_Fu1–Fu2_, is depicted in blue. Residue side chains involved in the interface are shown as sticks and labelled (atom colouring: dark blue, nitrogen; red, oxygen; yellow, sulphur). Dotted lines represent hydrogen bonds. A corresponding stereo figure with final electron density can be found in Supplementary Fig. S6. (**h**) The Met-finger pocket. Structural features are represented as in **g**. BR, basic region; PAD, protease-associated domain; SP, signal peptide; TM, transmembrane; TSR, thrombospondin-related domain.

Sequence analyses suggest a putative domain structure for the Rspo proteins comprising two furin-like cysteine-rich regions (Fu domains) plus a thrombospondin type 1 repeat domain^3^ (Fig. 1a). Our own and published data point to the involvement of the Fu domains in the potentiation of canonical Wnt signalling by Rspo proteins^1^,^19^,^20^,^21^ (Supplementary Fig. S1). We therefore engineered constructs to express the region spanning the two Fu domains of Rspo2 proteins from several species. We also generated secreted forms of the corresponding ZNRF3 and RNF43 ectodomains. The Rspo2_Fu1–Fu2_ and respective ZNRF3_ecto_ or RNF43_ecto_ molecules migrated together in gel filtration chromatography indicating high-affinity binding (data not shown), substantiating their ligand–receptor relationship. By using a combination of heavy atom and molecular-replacement-based phasing strategies, we determined multiple crystal structures for *Xenopus* (x) Rspo2_Fu1–Fu2_ (highest resolution 2.2 Å), xZNRF3_ecto_, zebrafish (z) ZNRF3_ecto_ and mouse (m) ZNRF3_ecto_, (highest resolutions 2.4, 1.6 and 2.0 Å, respectively), plus complexes comprising xZNRF3_ecto_–xRspo2_Fu1–Fu2_, mZNRF3_ecto_–mRspo2_Fu1–Fu2_, mZNRF3_ecto_–xRspo2_Fu1–Fu2_ and xRNF4F3_ecto_–xRspo2_Fu1–Fu2_ (at 2.1, 2.8, 2.4 and 2.7 Å, respectively; see Methods, Table 1 and Supplementary Table S1). In the following sections and figures, the highest resolution structures (Table 1 and Fig. 1) for the apo ligand, apo receptor and ligand–receptor complex will be used unless otherwise stated.

### Structure of the ZNRF3 ectodomain

The ZNRF3_ecto_ crystal structures revealed a distinctive variant of the protease-associated domain topology^22^. Two β-sheets (comprising β2, β1, β7, β3 and β4, β5, β6 strands, respectively; Fig. 1b) splay apart, accommodating an α-helix (αC) at the open edge; two additional α-helices (αA and αB) pack against the β4, β5 and β6 face of this distorted β-sandwich. A disulphide bridge, conserved across species, links two structurally elaborate loops, β3–β4 and β4–αA. The resultant single-domain structure is relatively compact. The crystal structures for apo xZNRF3_ecto_, zZNRF3_ecto_ and mZNRF3_ecto_ showed no major differences in the main chain conformation (Supplementary Fig. S2). Comparisons of ZNRF3_ecto_ structures for proteins crystallized in several different crystal lattices, or for crystals containing multiple copies in the asymmetric unit, consistently highlighted an acidic region (N105-E114; residue numbering is for mouse sequences unless otherwise stated) within the β3–β4 loop, the short αC–β7 loop and the extended β1–β2 hairpin as flexible elements of the fold (Supplementary Fig. S2). A search of the Protein Data Bank for structures with a similar topology yielded the ectodomain of GRAIL (gene related to anergy in lymphocytes) as the closest match (deposited as an unpublished crystal structure by J.R. Walker and colleagues, Structural Genomics Consortium; Protein Data Bank ID code 3ICU). GRAIL is a single-span transmembrane E3 ubiquitin ligase, which localizes to the endosomal compartment and promotes CD3 ubiquitinylation, acting as an essential regulator of T-cell tolerance^23^,^24^. The sequence identity between ZNRF3 and GRAIL ectodomains is low (13.4% for 127 residues); however, structural superposition revealed a shared three-dimensional fold consistent with a common evolutionary origin (r.m.s.d. 2.5 Å for 131 equivalent Cα pairs; Supplementary Fig. S3a).

**Figure 2:**
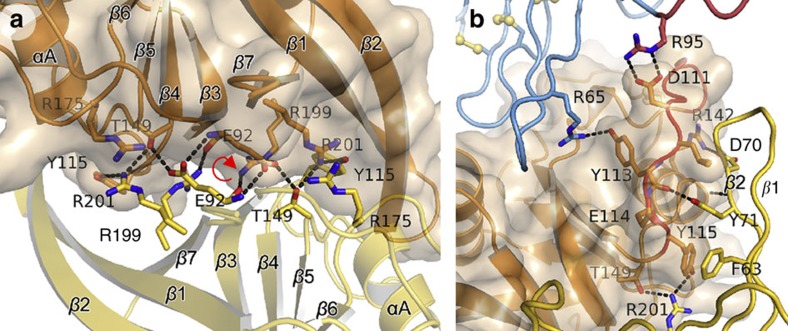
**Dimerization interface of xZNRF3**_**ecto**_. (**a**) View along the twofold axis away from the putative membrane. (**b**) xZNRF3_ecto_–xRspo_Fu1–Fu2_ complex with close-up view onto the β1–β2 hairpin arm (‘clamp’) embracing the respective other protomer. This interface is stabilized by binding of Rspo to ZNRF3 and subsequent structuring of the acidic region of the β3–β4 loop drawn in red. Residue numbers refer to mouse proteins.

Our crystallographic data provided independent structures for multiple copies of the ZNRF3 ectodomain in eight different crystal forms (Table 1 and Supplementary Table S1). All but two of these crystal structures reveal an extensive interface (average interface area 992±109 Å^2^; Supplementary Table S2) formed between two ZNRF3_ecto_ polypeptide chains. This dimer is conserved, and pairwise structural superpositions yielded r.m.s.d. values of <1.3 Å (for 275 equivalent Cα pairs; Supplementary Tables S3 and S4). The interaction is twofold symmetric; strands β3 and β7 of the ‘subunits’ abut face-to-face at the core of the dimer (Fig. 1c and 2a). The β1–β2 hairpin forms a second interface by reaching out to embrace helix αA and the β3–β4 loop in the opposing subunit (Fig. 2b). Intriguingly, these structural features interact in a parallel (*cis*) fashion consistent with ZNRF3 associating as a dimer on the cell surface.

### Structure of a signalling-competent fragment of Rspo2

In the crystal structure of the isolated Rspo_Fu1–Fu2_ protein (Table 1) the two Fu domains arrange sequentially to form a ladder-like structure of β-hairpins (Fig. 1d). Each Fu domain comprises three β-hairpins rigidified by four disulphide bridges (Fig. 1e), similar to the cysteine-rich regions found in members of the epidermal growth factor receptor family (Supplementary Fig. S3b). The connection between the Fu domains shows considerable rotational freedom, allowing a 50°–60° variation in the relative interdomain orientation (Supplementary Fig. S4). The N terminal of the two domains, Fu1, is distinguished by the extension of the second β-hairpin (Fig. 1d,e). This prominent loop presents a solvent exposed methionine (M68) at its tip, which we term the ‘Met-finger’.

### Structure of liganded complexes of ZNRF3_ecto_ and RNF43_ecto_

**Figure 3:**
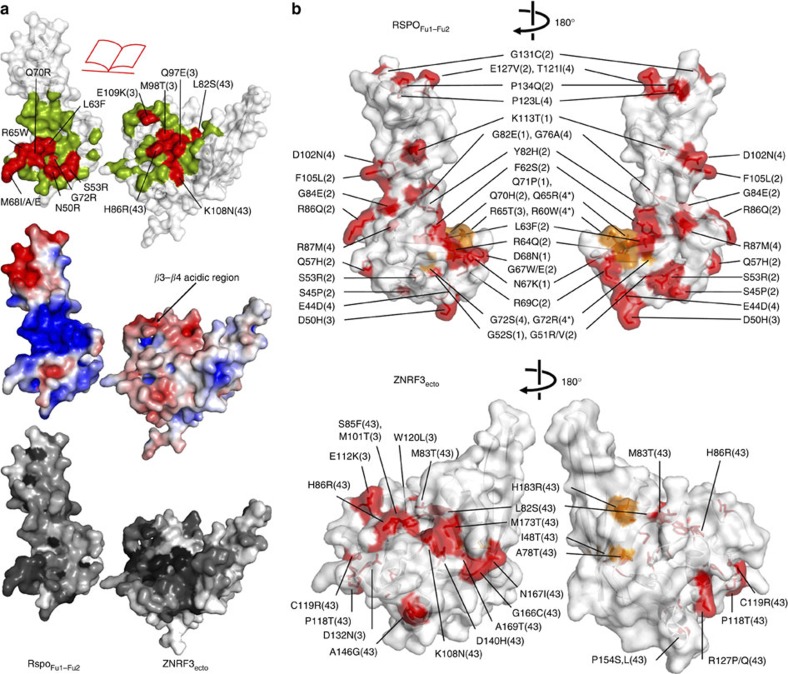
Characteristics of the ZNRF3 dimer and Rspo–ZNRF3 complex interfaces. (**a**) An open book view of the ZNRF3–Rspo interface. The surface contributing to the interface is coloured green on ZNRF3_ecto_ and Rspo_Fu1–Fu2_; within this, surface mutants tested in this study are highlighted in red (top). Rspo and ZNRF3_ecto_ coloured by electrostatic surface potential from red (acidic) to blue (basic) (middle). Sequence conservation across species coloured from white (not conserved) to black (conserved). (**b**) Disease-related mutations are plotted onto the molecular surface of Rspo (top) and ZNRF3/RNF43 (bottom), and are concentrated at the Rspo–ZNRF3/RNF43 interaction interface. Tumour-associated missense mutations derived from the cosmic database ( http://cancer.sanger.ac.uk/cancergenome/projects/cosmic/) are shown in red and missense mutations causal for congenital anonychia on *RSPO4* are shown in orange. Sites in orange on ZNRF3 are mutations of RNF43 that map to the dimer interface of ZNRF3. Numbers 1–4 in parentheses indicate mutations found in RSPO1 to RSPO4 (top). Number 3 and 43 in parentheses indicate mutations found in ZNRF3 and RNF43, respectively (bottom).

The crystal structures of the ZNRF3_ecto_–Rspo2_Fu1–Fu2_ and RNF43_ecto_–Rspo2_Fu1–Fu2_ complexes (Table 1 and Supplementary Table S1) revealed a 1:1 interaction between Fu1 of the Rspo2_Fu1–Fu2_ and a single ZNRF3_ecto_ or RNF43_ecto_ chain (Fig. 1f). As will be discussed below, all complex structures, except for the RNF43 complex, reveal a conserved 2:2 stoichiometry (Supplementary Fig. S5a). The interaction interface between Rspo2_Fu1–Fu2_ and its two receptors RNF43 and ZNRF3 is essentially the same (Supplementary Fig. S5b). It involves an interface area of 990±105 Å^2^ (Fig. 1g,h, Fig. 3, Supplementary Fig. S6 and Supplementary Table S2). Because of the availability of higher resolution data and multiplicity of data sets, we will first focus on the Rspo2–ZNRF3 interaction. Neither the Rspo2_Fu1–Fu2_ nor the ZNRF3_ecto_ dimer show major conformational changes on complex formation (Supplementary Fig. S5). The first, extensive, area of interaction involves hydrophobic interactions interspersed with hydrophilic (complementarily charged) patches contributed by the first two β-hairpins of the Rspo Fu1 and the region immediately carboxy-terminal to the β3 strand of ZNRF3_ecto_ (Fig. 1g). The Met-finger at the tip of the second β-hairpin of Fu1 nestles into a pocket formed between the β3 strand and the αC–β7 loop of the ZNRF3_ecto_, which is lined with hydrophobic residues (I95, I191, V192, A198; Fig. 1h). The αC–β7 loop is a flexible region in the unliganded ZNRF3_ecto_ crystal structures and moulds to interface the Rspo_Fu1–Fu2_ M68 in the complex. Overall, the ZNRF3_ecto_ dimer structure appears less flexible in the complex structures compared with the unliganded structures. The acidic region of the β3–β4 loop (immediately adjacent to C104 of the disulphide bridge) becomes more ordered in the ligand-bound ZNRF3_ecto_ structures (Supplementary Fig. S2), probably as a result of electrostatic interactions with a positively charged patch on Rspo Fu1 (Fig. 3a).

### Biophysical and cellular analyses support the structure data

**Figure 4:**
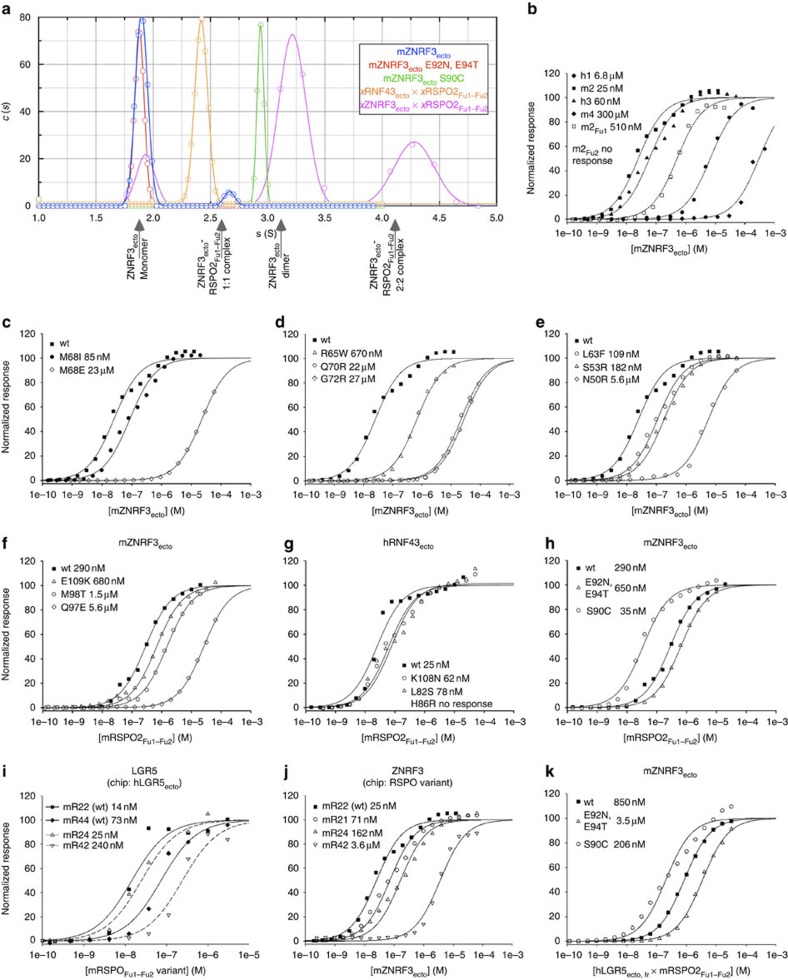
Biophysical characterization of the ZNRF3ecto dimer and interface mutants. (**a**) Sedimentation velocity experiments. A plot of *c*(*s*) (in arbitrary units) against s (in svedbergs). Shown in each case are individual data points and the fit of an appropriate number of Gaussian distributions. All samples were adjusted to a concentration of 350 μM. Also shown arrowed are the expected sedimentation coefficients for the different complexes observed in the crystal structures as predicted using HYDROPRO (see Methods). (**b**–**h**) SPR experiments using mZNRF3_ecto_ (**b**–**e**) or mRspo2_Fu1–Fu2_ (**f**–**h**) as analyte and interface mutants/variants as immobilized ligands. (**b**) mZNRF3_ecto_ binds to mRspo2_Fu1–Fu2_ (I39-G144) and retains high affinity to Fu1 (I39-R95) but not to Fu2 (A94-G144). Fu1–Fu2 polypeptides of human or mouse homologues (hRspo1: I32-S143, hRspo3: R32-H147, mRspo4: T29-Q136) bind with different affinity to mZNRF3_ecto_. (**c**) Mutations of the Met-finger impact affinity. (**d**) Anonychia mutations of RSPO4 introduced to mRspo2_Fu1–Fu2_ drastically impair binding. (**e**) Three additional interface mutants of which two (L63F and S53R) have been found in tumour tissues. (**f**) As the immobilized ligand mZNRF3 binds with lower affinity to the mRspo_2Fu1–Fu2_ analyte. Of the three interface mutants, two (E109K and M98T) have been identified in tumour tissues. (**g**) Three interface mutants of hRNF3_ecto_ have been identified in tumours, one of which completely disrupts binding. (**h**) Binding of mRspo2_Fu1–Fu2_ to ZNRF3_ecto_ dimer interface mutants. (**i**) Binding of mRspo2_Fu1–Fu2_, mRspo4Fu1–Fu2 and chimeras to hLGR5_ecto_. Single dilution series. (**j**) Binding of Rspo_Fu1–Fu2_ chimeras to ZNRF3_ecto_. (**k**) Binding of the preformed hLGR5_ecto,lr_–Rspo2_Fu1–Fu2_ complex to ZNRF3_ecto_ dimer interface mutants.

**Figure 5:**
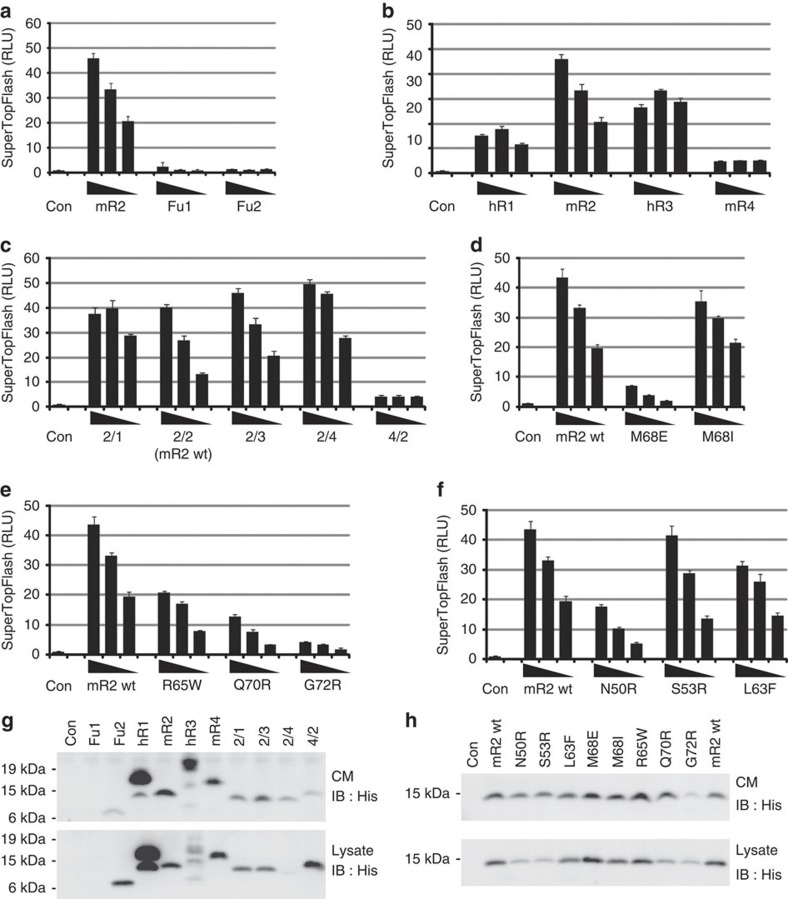
Activation of the Wnt pathway assayed by the SuperTopFlash reporter. (**a**–**f**) Co-transfected decreasing doses (25, 5 and 1 ng) of His-tagged R-spondin constructs used for SPR experiments in Fig. 4. Error bars represent s.d. from three replicates. (**g**,**h**) Western blots showing expression levels of the His-tagged R-spondin constructs from whole-cell lysate and conditioned media (CM). Expression for mRspo2 Fu1-His was poor and below the level of detection; however, the individual Fu1 domain from RSPO1 was detected by western blotting and produced identical results (Supplementary Fig. S1b,c). RLU, relative luciferase units.

Analytical ultracentrifugation results are consistent with ZNRF3ecto dimer formation and analyses of several ZNRF3-Rspo2 and RNF43-Rspo2 interface mutants (Figs 1g,h and 3) using surface plasmon resonance (SPR)-binding assays confirm the crystallographically determined complex structures (Fig. 4). The single-domain protein mRspo2_Fu1_ still bound mZNRF3_ecto_ with high affinity, whereas no detectable binding was measured for mRspo2_Fu2_ (Fig. 4b). Consistent with the high level of surface residue conservation at the interface (Fig. 3a), the Fu1–Fu2 repeats for all four members of the Rspo family showed binding to ZNRF3_ecto_ in SPR assays (Fig. 4b). However, the fine-grained differences in the interface-forming residues did impact on the binding affinities; the stronger binding of Rspo2_Fu1–Fu2_ versus Rspo1_Fu1–Fu2_ and Rspo4_Fu1–Fu2_ appeared to be conferred, in part, by the substitution of isoleucine for methionine at the tip of the second β-hairpin (Fig. 4c). Previously reported genetic and cancer-associated mutations further corroborate the functional significance of the ZNRF3_ecto_–Rspo2_Fu1–Fu2_ interface as the generic interaction mode for Rspo1–4 and ZNRF3/RNF43 (Fig. 4d–g). For example, in Rspo4, the equivalent of the R65W, Q70R and G72R mutations have been reported in inherited anonychia^8^,^9^,^10^. From an analysis of the interaction interface, it is obvious that these mutations are not compatible with ZNRF3/RNF43 binding (Fig. 1g). Functional assays that measure Rspo signalling activity in cells further support the significance of the Rspo–ZNRF3 interface (Fig. 5 and Supplementary Fig. S7). For example, a Met-finger mutation, M68E, which profoundly compromised Rspo–ZNRF3 interaction in SPR assays, exhibited much weaker signalling activity, whereas a conserved substitution, M68I, showed slightly reduced binding to ZNRF3 and relatively normal (or slightly reduced) signalling capacity (Fig. 5d). Other interface mutants in Fu1, including the anonychia-associated mutations R65W, Q70R and G72R, as well as N50R, each exhibited weakened signalling ability that correlated with reduction in binding to ZNRF3 (Fig. 5e).

### Dimerization propensity of ZNRF3 versus monomeric RNF43

For all ZNRF3_ecto_–Rspo2_Fu1–Fu2_ complex structures we determined (from five different combinations of species and crystal forms), the dimer found in most of the unliganded ZNRF3_ecto_ crystal structures reoccurs (Fig. 1f, Supplementary Fig. S5 and Supplementary Table S2). The overall assembly thus comprises a 2:2 complex of ZNRF3_ecto_–Rspo2_Fu1–Fu2_. The 2:2 complex resembles a crab with the ZNRF3_ecto_ dimer forming the body from which the two Rspo2_Fu1–Fu2_ ligands diverge, without interacting with each other, as the pincers. In contrast, our single structure of RNF43 in complex with Rspo2_Fu1–Fu2_ displays no dimeric architecture.

Dimerization of ZNRF3_ecto_ is weak in solution and the protein did not behave as a dimer in gel filtration. Still, a propensity of ZNRF3_ecto_ to dimerize was evident from analytical ultracentrifugation data. Broad peaks of ZNRF3_ecto_ from ultraviolet absorbance data were highly indicative of a rapid equilibrium of self-association. In sedimentation velocity plots using the faster interference optics traces of a dimer could be detected (Fig. 4a). This rapid dimerization was not observed when a glycosylation site E92N, E94T was engineered into the dimerization interface observed in the crystal structures. Formation of the observed crystallographic ZNRF3 dimer in solution is further supported by the observation of almost quantitative spontaneous crosslinking of the S90C variant of mZNRF3_ecto_ that introduces a cysteine close to the dimer symmetry axis (Supplementary Fig. S8). A crystal structure of this variant at 2.1 Å shows that this mutation and crosslinking is easily accommodated, requiring only minor backbone distortions (Supplementary Table S1 and Supplementary Fig. S8d).

Formation of the ZNRF3_ecto_–Rspo2_Fu1–Fu2_ complex also leads to increased dimerization in solution (Fig. 4a). An explanation for this is found by careful analysis of the crystal structures. As outlined before, ligand binding leads to a structuring of the acidic region of the β3–β4 loop (red in Figs 1b,c and 2b). This same region of the β3–β4 loop also interacts with the β1–β2 hairpin in the opposing subunit of the ZNRF3_ecto_ dimer (Fig. 2b). Notably, the β1–β2 hairpin shows less conformational variation in the liganded ZNRF3_ecto_ dimer structures, always maintaining a tight embrace (Supplementary Figs S2b and S5a), consistent with the Rspo2_Fu1–Fu2_ interactions contributing an indirect stabilizing effect on the dimer via the β3–β4 loop. This stabilizing effect provides an explanation for our results showing that Rspo2_Fu1–Fu2_ bound weaker to monomerized mZNRF3_ecto_ E92N, E94T but stronger to predimerized mZNRF3_ecto_ S90C than to the wt mZNRF3_ecto_ (Fig. 4h).

No dimer is observed in solution for RNF43_ecto_, even after binding to mRspo2_Fu1–Fu2_ (Fig. 4a), and we also see only a minor propensity for spontaneous cysteine crosslinking of the P77C (corresponding to S90C of mZNRF3_ecto_) variant of hRNF43_ecto_ (Supplementary Fig. S8b). We note that the residues involved in the dimerization interface of ZNRF3, albeit conserved within the ZNRF3 family, are not conserved between ZNRF3 and RNF43 (Supplementary Fig. S5c). Furthermore, two glycosylation sites exist in RNF43 and map to the acidic region of the β3–β4 loop and the β1–β2 clamp (Supplementary Figs S2 and S5). These sites are not resolved in the RNF43 complex but might sterically hamper dimerization.

### Rspo interacts with LGRs via Fu2

**Figure 6:**
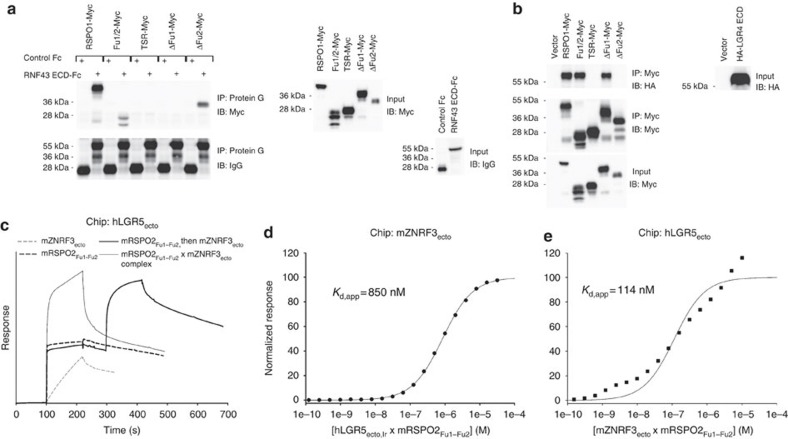
The LGR–Rspo–ZNRF3/RNF43 ternary complex. (**a**) RNF43 interacts with Fu1 of human Rspo1. The secreted RNF43 ectodomain co-immunoprecipitated Rspo1 and its derivatives, Rspo1_Fu1–Fu2_ and Rspo1_ΔFu2_, but neither RSPO1_ΔFu1_ nor Rspo1_TSR_ in conditioned media (CM; left). RNF43 is IgG-tagged, whereas Rspo1 and derivatives are Myc-tagged, and their secretion levels in CM were also examined (right). (**b**) LGR4 interacts with Fu2 of Rspo1. The secreted LGR4 ectodomain was co-immunoprecipitated by Rspo1 and its derivatives, Rspo1_Fu1–Fu2_ and Rspo1_ΔFu1_, but by neither Rspo1_ΔFu2_ nor Rspo1_TSR_ in CM (left). Secreted LGR4 is HA-tagged and its secretion in CM was examined as were Rspo1 and derivatives (right). (**c**) Step-by-step ternary complex assimilation. hLGR5ecto (R32-G557) was immobilized on an SPR chip, followed by injections of 10 μM solutions of mZNRF3_ecto_, mRSPO2_Fu1–Fu2_, mRSPO2_Fu1–Fu2_ followed by mZNRF3_ecto_ or a preformed mZNRF3_ecto_ × mRSPO2_Fu1–Fu2_ complex. mZNRF3_ecto_ shows some direct interaction with LGR5 characterized by a slow on-rate (thin dashed line). Binding is much faster if LGR5 is first saturated with mRSPO2 _Fu1–Fu2_ (thick solid line). Similar responses are observed when a 1:1 complex of mRSPO2_Fu1–Fu2_ × mZNRF3_ecto_ is injected. (**d**) Saturation of immobilized mZNRF3_ecto_ with the hLGR5_ecto_ × mRSPO2_Fu1–Fu2_ complex that was stable in gel filtration. lr, loop removed: A488-H537→NGNNGD. (**e**) Saturation of immobilized hLGR5_ecto_ with the mZNRF3_ecto_ × mRSPO2_Fu1–Fu2_ complex that was stable in gel filtration. Single dilution series.

**Figure 7:**
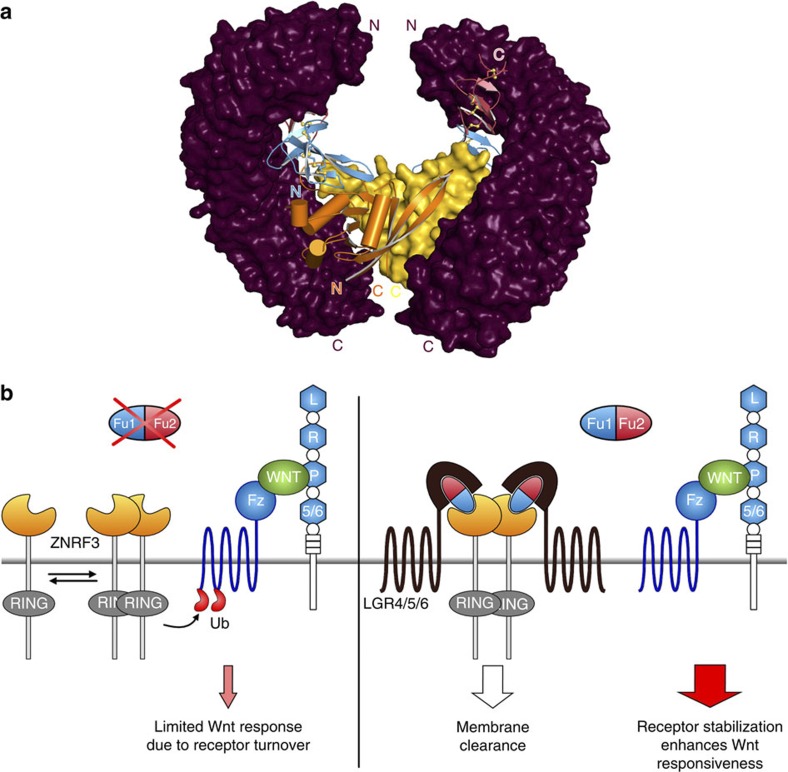
**Modelling of a ternary 2:2:2 LGR**_**ecto**_**–Rspo**_**Fu1–Fu2**_**–ZNRF3**_**ecto**_ **complex and its implication for signalling.** (**a**) The hLGR5_ecto_ × hRSPO1_Fu1–Fu2_ × mZNRF3_ecto_ 2:2:2 complex was generated by superposing the ternary hLGR5_ecto_ × RSPO1_Fu1–Fu2_ × hRNF43_ecto_ complex^25^ (Protein Data Bank ID code 4KNG) onto the mZNRF3_ecto_ dimer from the mRSPO2_Fu1–Fu2_ complex. No clashes are observed. Glycosylation sites of LGR5 all point into the periphery of the shown complex. (**b**) A model for regulation of Wnt signalling by RSPO and its receptors based on our results and those by Hao *et al.*^16^ The schematic model takes into account the different binding sites of LGRs and ZNRF3/RNF43 on Rspos as determined by us and others^25^,^26^,^27^,^28^.

Fu2, similar to Fu1, is essential for Rspo signalling function^1^ (Supplementary Fig. S1). We therefore suspected that Fu2 might be involved in binding to other components of the Rspo receptor complex, such as LGR4/5/6. Indeed, although Rspo1, Rspo1_Fu1–Fu2_ and Rspo1_ΔFu2_ were each co-immunoprecipitated with RNF43, confirming that Fu1 is critical for binding to ZNRF3/RNF43, Rspo1, Rspo1_Fu1–Fu2_ and Rspo1_ΔFu1_ each immunoprecipitated LGR4, indicating that Fu2 is the primary binding site for LGR receptors (Fig. 6a,b). Consistent with these co-immunoprecipitation data, Rspo2_Fu1–Fu2_ simultaneously bound to ZNRF3 and LGR5 in SPR binding assays (Fig. 6c–e) with the monomerized ZNRF3_ecto_ binding weaker and the dimerized S90C variant binding stronger to a preformed LGR5_ecto_–Rspo_Fu1–Fu2_ complex (Fig. 4k). Our data therefore support the model of a ZNRF3/RNF43–Rspo–LGR4/5/6 complex assembled through ZNRF3/RNF43–Rspo_Fu1_ and Rspo_Fu2_–LGR4/5/6 interactions (Fig. 7).

### Rspo–ZNRF3/RNF43 interaction determines signalling potency

Although there is a clear requirement for both Furin domains in Rspo ternary complex formation and functional activation of the Wnt pathway, our results point to the ability of Rspo_Fu1_ to recruit ZNRF3/RNF43 as the major determinant of activity (for example, Figs 4b and 5b). In biophysical assays, wild-type mRspo2 and -4 proteins, as well as their Fu1/Fu2 chimeras, bound with nanomolar affinity to hLGR5_ecto_ (Fig. 4i), further suggesting that engagement of LGR is not the efficiency-determining step. Cellular assays using Rspo2 Fu1/Fu2 chimeras showed that a Fu1 repeat from a ‘strong’ Rspo (that is, Rspo2 or -3) was sufficient to induce a higher Wnt response (Fig. 5c). On the other hand, in spite of an 80-fold (300 μM >3.6 μM) increase in binding efficiency to ZNRF3_ecto_, replacement of Fu2 of Rspo4 by that of a ‘strong’ Rspo was not able to enhance Wnt signalling when expressed at comparable levels (Fig. 5c). Hence, it can be concluded that functional efficiency of the four Rspo ligands is largely based on their ability to recruit ZNRF3 or RNF43 via Fu1 into a complex with LGRs.

## Discussion

In combination, the structural, biophysical and cell-based studies we report here for the ZNRF3/RNF43–Rspo system reveal two modes of interaction: receptor–ligand and receptor dimer. For the ligand–receptor mode, our data define a generic architecture for the interaction between the Rspo ligands, and the ZNRF3 and RNF43 transmembrane E3 ubiquitin ligases that is conserved across evolution from fish to human. Indeed, the differences in binding affinities, from highest affinity for Rspo2 to lowest for Rspo4, appear to mirror the trend in biological activity of the four Rspo proteins (reviewed in ref. 3). Our results highlight the role of the Fu1 domain of the Rspo protein in ZNRF3/RNF43 binding. Both Fu domains together have been implicated in Rspo signalling. The primary role of Fu1 in ZNRF3/RNF43 binding leaves a substantial surface available for the Rspo to mediate formation of a three component complex involving ZNRF3/RNF43, Rspo and LGR4/5/6 as postulated^16^. Indeed, our co-immunoprecipitation results focus LGR4/5/6-binding activity onto Fu2, consistent with Rspo proteins acting as complex assemblers (Fig. 7). Whilst we were preparing our paper for publication, several crystal structures of Rspo1_Fu1–Fu2_ in complex with LGR4/5_ecto_ and a single Rspo1_Fu1–Fu2_–LGR5_ecto_–RNF43_ecto_ complex were reported^25^,^26^,^27^,^28^, which fully support this notion.

Unexpectedly, our analyses reveal a dimerization mode for ZNRF3_ecto_. The conservation of ZNRF3 ectodomain dimerization across evolution from fish to mammals suggests that this interaction has some role in the mechanism of action of ZNRF3. It is also noteworthy that three cancer-associated mutations reported for RNF43 map to the corresponding dimer interface observed in the ZNRF3_ecto_ crystal structures (Fig. 3b), suggesting the characteristics of this surface have functional relevance in RNF43 as well. Many members of the E3 RING ubiquitin ligase superfamily have been reported to require dimerization for function (reviewed in ref. 29), a conclusion supported by recent insights into the mechanism of action of the RING ligase RNF4 (ref. 30). In ZNRF3, the ectodomain may, alongside cytoplasmic regions, contribute to functionally essential RING domain dimerization. However, neither our biophysical measurements nor our structural data for an RNF43 ectodomain in complex with Rspo2 provide any evidence of a similar dimerization mode for RNF43; a finding that argues against ectodomain dimerization having a central role in ligase activity. The newly reported structure of the 1:1:1 complex of Rspo1–LGR5–RNF43 also reveals no RNF43 dimer^25^. Intriguingly at the level of a simple modelling exercise, the ternary complex architecture appears compatible with ZNRF3 dimerization (Fig. 7). All of the currently available structures are compatible with a dimeric ZNRF3 as reported here. However, we note that none of the reported crystallographic LGR4/5_ecto_ dimers would be compatible with simultaneous binding of Rspo proteins to both LGR4/5/6 and ZNRF3/RNF43. Our observation is suggestive of Rspo functioning to sequester ZNRF3 dimers into a complex with LGR4/5/6. Thus, we may speculate that the difference in oligomeric state of the ZNRF3 and RNF43 ectodomains points to some yet to be ascertained difference in their function or regulation. We note that sequence conservation of the ectodomain within the ZNRF3 subfamily is far greater than that within the RNF43 subfamily (Supplementary Table S5), supporting the notion that for ZNRF3 function or regulation additional features such as ligand-induced dimerization may be important.

ZNFR3 and RNF43, alongside the Rspo proteins, have emerged as a system with significant therapeutic potential for a number of pathological processes. The insights into molecular mechanism presented here open up new avenues to explore for possible manipulation of this system.

## Methods

### Large-scale expression of ZNRF3_ecto_ and RSPO_Fu1–Fu2_

Synthetic complementary DNA clones for ectodomains of mouse and zebrafish ZNRF3 and *Xenopus* RNF43 were obtained from Invitrogen/Geneart (Germany). All other template cDNAs were from the I.M.A.G.E. library.

*Xenopus*, mouse and zebrafish ZNRF3 ectodomains (residues E25-D191, K53-L205 and K30-R181, respectively), *Xenopus* RNF43 ectodomain (T28-D192), as well as *Xenopus* and mouse Rspo2_Fu1–Fu2_ constructs (residues G35-D143, N37 or I39-G144) were cloned into the pHLsec vector^31^ that encodes for a C-terminal His6-tag (His10-tag for mRSPO2_Fu1–Fu2_). Proteins were expressed separately or after co-transfection in HEK293T cells seeded into roller bottles. For preparation of seleno methionine (SeMet)-labelled xRSPO2_Fu1–Fu2_, the cells were washed 24 h after transfection with PBS (2 × 35 ml per roller bottle) and the medium was changed to methionine-free DMEM complemented with 2% dialysed fetal bovine serum and 40 μg ml^−1^ SeMet. After 4–8 days expression, the medium was collected and cleared by centrifugation and filtration. The buffer was exchanged to 10 mM Tris/HCl, pH 8.0, 500 mM NaCl using hollow-fibre ultrafiltration. Proteins were purified using Ni2^+^-charged 5 ml HisTrap FF columns from GE. Before sample loading, 25 mM imidazole was added to suppress unspecific binding. The elution buffer contained 1 M imidazole in binding buffer. Individual proteins were subjected to gel filtration on S200 16/60 pg columns (GE Healthcare) equilibrated with 10 mM HEPES/NaOH, pH 7.5, 150 mM NaCl. ZNRF3_ecto_–RSPO2_Fu1–Fu2_ complexes were obtained by coexpression or by mixing of equimolar amounts followed by gel filtration. Because of solubility issues of the complexes, the NaCl concentration of the gel filtration buffer was increased to 250 mM.

### Crystallization and data collection

Concentrated proteins were subjected to sitting drop vapour diffusion crystallization trials employing a Cartesian Technologies pipetting robot, and usually consisted of 100–300 nl protein solution and 100 nl reservoir solution^32^. Crystal form I of apo xRSPO2_Fu1–Fu2_ appeared in 100 mM Bis-Tris/HCl, pH 6.3, 200 mM ammonium sulphate (AS) and 1.2 M tartrate, pH 7.5, at a protein concentration of 37.5 mg ml^−1^. Crystals of form II appeared in 100 mM cacodylate and 1 M sodium citrate, final pH 7, at a protein concentration of 24 mg ml^−1^. zZNRF3_ecto_ crystallized in 20% (w/v) PEG3350, 200 mM CaCl_2_ (form I) or 25% (w/v) PEG3350, 100 mM Bis-Tris, pH 5.5 (form II). Crystals of mZNRF3_ecto_ appeared at sample concentrations of 49 mg ml^−1^ in 25% (w/v) PEG3350, 200 mM MgCl_2_ and 100 mM Tris, pH 8.5, (form I); 20% (w/v) PEG3350, 5% (w/v) low-molecular-weight polyglutamic acid and 100 mM Tris, pH 7.8, (form II); 20% (w/v) PEG8000, 200 mM MgCl_2_ and 100 mM Tris, pH 8.5 (form III); or crystallized alone out of a complex sample with bovine RSPO2_Fu1–Fu2_ at 16.2 mg ml^−1^ in 0.5 M Li_2_SO_4_ and 10% (w/v) PEG8000 (form IV). The S90C variant of mZNRF3_ecto_ crystallized in 45% (v/v) 2-methyl-2,4-pentanediol, 200 mM ammonium acetate and 100 mM Bis-Tris, pH 5.5, at a concentration of 38.5 mg ml^−1^. Apo xZNRF3_ecto_ crystallized at 25 mg ml^−1^ in 20% (w/v) PEG3350, 100 mM Bis-Tris propane, pH 6.5, 200 mM NaBr (form I), and 20% (w/v) PEG3350 and 0.200 M NaCl (form II). Crystals composed of the complex of mZNRF3_ecto_ and mRSPO2_Fu1–Fu2_ were obtained at a concentration of 18 mg ml^−1^ in 1.8 M AS, 100 mM Bis-Tris, pH 6.5, 2% (v/v) PEGMME550. Crystals of the mixed species complexes of mZNRF3_ecto_ and SeMet xRSPO2_Fu1–Fu2_ appeared in 25% (w/v) PEG4000, 200 mM NaCl, 100 mM HEPES/NaOH, pH 7.5 (form I), and 20% (w/v) PEG3350, 200 mM sodium citrate, 100 mM Bis-Tris propane, pH 6.5 (form II). xZNRF3_ecto_–xRSPO2_Fu1–Fu2_ complexes crystallized in 20% (w/v) PEG3350, 200 mM (NH_4_)F (form I) and 20% (w/v) PEG6000, 100 mM MES, pH 6.0 (form II). The complex of xRNF43_ecto_ and xRSPO2_Fu1–Fu2_ was crystallized in condition A1 of the PACT premier screen from Molecular Dimensions. For cryoprotection, crystals were transferred to mother liquor supplemented with 1.7 M sodium malonate, pH 7 (both apo xRSPO2_Fu1–Fu2_ crystals), with AS to 3 M (mouse/mouse complex), or with PEG200 to achieve total (PEG, polyethylene glycol)>30% (all other ZNRF3 apo and complex crystals) by incrementally adjusting the concentration of the cryoprotectant. Crystal were then flash-cooled by dipping into liquid nitrogen. The xRNF43_ecto_–xRSPO2_Fu1–Fu2_ complex crystal was frozen directly and showed strong ice rings. Diffraction data were collected at DIAMOND synchrotron light source at the beamlines i02, i03, i04 and i24. Crystal forms II and III of apo mZNRF3_ecto_ had been soaked with a platinum compound, but showed only low binding of heavy atoms.

### Structure determination

The structure of xRspo2_Fu1–Fu2_ was solved using highly redundant single-wavelength anomalous dispersion (SAD) data from a Pt(IV)-soaked crystal that diffracted to 3.2 Å (Table 1). Ten strong anomalous sites could be identified by AUTOSHARP^33^. Refinement and subsequent density modification with SOLOMON lead to clearly interpretable electron density. A partial model obtained from BUCCANEER^34^ was used to solve the high-resolution structure. The model was improved with iterative rounds of manual building in COOT^35^ and refinement in REFMAC5 (ref. 36). The structure of mZNRF3_ecto_ in complex with SeMet-labelled xRSPO2_Fu1–Fu2_ was solved from SAD data collected at the Se K-absorption edge. Albeit only one component of the complex was labelled and the complex being crystallized in the low-symmetry space group P1, the Se atom substructure (four sites) could be identified by PHENIX HYSS^37^ from average redundancy data (Supplementary Table S1). An initial model generated by AUTOSOL was used to solve the high-resolution mZNRF3_ecto_ structure (Supplementary Table S1). All other structures were solved by molecular replacement with PHASER^38^ and completed by manual rebuilding in COOT and refinement with REFMAC5. Models were validated with MOLPROBITY^39^. Superpositions were performed within CCP4 or COOT using the SSM algorithm. Electrostatics potentials were generated using APBS^40^, surface sequence conservation was calculated using CONSURF^41^ and interface areas of proteins were calculated using the PISA web server^42^. Figures were produced in PYMOL and assembled in PHOTOLINE32.

### Analytical ultracentrifugation

xZNRF3_ecto_–xRSPO2_Fu1–Fu2_ and xRNF43_ecto_–xRSPO2_Fu1–Fu2_ complexes and apo mZNRF3_ecto_ variants at 350 μM in 10 mM HEPES/NaOH, 250 mM NaCl were subjected to sedimentation velocity experiments at 20 °C using an Optima Xl-I analytical ultracentrifuge (Beckman) with 3 mm or 12 mm double sector centerpieces in an An-60 Ti rotor (Beckman) at 40,000 r.p.m. Sedimentation was monitored by ultraviolet absorption at 300 nm and by Rayleigh interference. Data were analysed using SEDFIT operating in *c*(*s*) and *c*(*s*,*f*/*f*_o_) modes (with a frictional coefficient range of 1–2 in the latter case and a resolution in s of 100)^43^. The resulting sedimentation coefficient distributions were plotted using ProFit (Uetikon am See, CH). The crystal structures were modelled hydrodynamically using the programme HYDROPRO^44^.

### SPR equilibrium binding studies

Affinity between variants of mZNRF3_ecto_, human RNF43_ecto_ and mRSPO2_Fu1–Fu2_ was measured at 25 °C in 10 mM HEPES/NaOH, pH 7.5, 150 mM NaCl, 0.005% Tween20 using a Biacore T200 machine (GE Healthcare). Synthetic DNA corresponding to mZNRF3(K53-L205), hRNF43(Q44-L188) and mRSPO2(I39-G144), as well as variants thereof, was obtained from Invitrogen/Geneart (Germany) and cloned into a variant of the pHLsec vector encoding a C-terminal recognition sequence for the *Escherichia coli* BirA enzyme. Biotinylation at this sequence tag was performed as described^45^. Experiments were performed as described before^46^, with the biotinylated variants immobilized to the chip surface precoupled with approximately 10,000 resonance units (RU) of streptavidin. Immobilized protein amounts varied between 350 and 1,000 RU (1 experiment with 1,650 RU). The amount of immobilized protein did not seem to strongly influence the binding model. After each injection of analyte, the chip surface was regenerated with 2 M MgCl_2_, 10 mM HEPES/NaOH, pH 7.5 (RSPO coupled), 100 mM phosphate, pH 3.7, 2 M NaCl and 1% (v/v) Tween20 (RNF43 or ZNRF3 coupled) or 25% ethylene glycol, 2 M NaCl, 100 mM HEPES/NaOH, pH 7.5, 1% Tween20 (LGR coupled) to return to baseline levels. Data were fitted to a Langmuir adsorption model *B*=*B*_max_*C*/(*K*_d_+*C*), where *B* is the amount of bound analyte and *C* is the concentration of analyte in the sample. Data were then normalized to a maximum analyte-binding value of 100. Unless stated otherwise, data points correspond to average from two independent dilution series.

### Co-immunoprecipitation binding assays

Full-length human RSPO1-Myc (1–263), and domains of Fu1/Fu2 (1–147) and TSR (1–20, 144–263), were originally reported in ref. 47. On the basis of the disulphide bond pattern resolved in the crystal structure, new individual Fu domain deletions were generated: deltaFurin1 (del 39–94; Rspo1_ΔFu1_) and deltaFurin2 (del 97–142; Rspo1_ΔFu2_). For immunoprecipitation, conditioned medium from HEK293T cells transfected using FugeneHD with RSPO1-Myc, Furin1/2-Myc, TSR-Myc, deltaFurin1-Myc or deltaFurin2-Myc was mixed with conditioned medium from cells transfected with IgG, Human RNF43 ECD-IgG (1–198) or mouse HA-Lgr4 ECD (from ref. 11), and incubated at 4 °C overnight. The mixture was then incubated with protein G-agarose beads for 2 h at 4 °C and washed with buffer (150 mM NaCl, 1 mM EDTA, 2.5 mM EGTA, 10% glycerol, 0.1% Tween20 with protease inhibitors). Protein was eluted using 2 × SDS sample buffer and separated by SDS–PAGE. Western blotting was performed by using horseradish peroxidase-conjugated anti-human IgG (Calbiochem), anti-HA or anti-c-Myc. For co-immunoprecipitation assays, anti-Myc (9E10, Santa Cruz) was used at a 1:100 concentration. For western blots, primary antibodies were diluted 1:1000 and secondary antibodies were diluted 1:10000 from stocks.

### Activity assays

To assess Rspo activation of the Wnt signalling pathway, a traditional dual-luciferase assay consisting of the Wnt-responsive SuperTopFlash reporter (normalized to a control promoter driving Renilla luciferase) was used as previously described^46^. Mammalian cell transfections were done in HEK293T (ATCC CRL-11268) cells and performed in triplicate for each sample condition. Cells were plated at 1 × 10^5^ per ml in 24-well plates and transfected the following day with a total of 200 ng of DNA per well (50 ng SuperTopFlash, 10 ng TK-Renilla, experimental expression vectors and balanced with empty vector). Lysates were collected 36 h post transfection and used with the Dual-luciferase reporter system (Promega). Firefly and Renilla luciferase activity was measured using the Wallac 1420 multilabel counter in 96-well plates. Normalized data expressed in relative luciferase units was averaged from triplicate assays and error bars reflect s.d. Representative results are shown from one of multiple independent experiments.

## Author contributions

All authors contributed to the design of the project, data analysis and preparation of the manuscript. M.Z. cloned, purified and performed SPR and AUC experiments on Rspo proteins and ZNRF3, crystallized and solved the individual and complex structures. C.K. contributed to cloning, protein purification, SPR and diffraction data collection. R.J.C.G. contributed to AUC data collection and analysis. B.T.M., Y.X. and M.C. cloned and performed functional assays and co-immunoprecipitation for Rspo proteins.

## Additional information

**Accession codes:** Coordinates and structure factors for zZNRF3_ecto_, mZNRF3_ecto_, xZNRF3_ecto_, xRspo2_Fu1–Fu2_, mZNRF3_ecto_–mRspo2_Fu1–Fu2_, mZNRF3_ecto_–xRspo2_Fu1-Fu_, xZNRF3_ecto_–xRspo2_v_ and xRNF43_ecto_–xRspo2_Fu1–Fu2_ crystal structures have been deposited in the Protein Data Bank with the succession numbers 4C84, 4C85, 4C86, 4C8A, 4C8C, 4C8F, 4C8P, 4C8T, 4C8U, 4C8V, 4C8W, 4C99, 4C9A, 4C9E, 4C9R, 4C9U and 4C9V as also given in Table 1 and Supplementary Table S1.

**How to cite this article:** Zebisch, M. *et al.* Structural and molecular basis of ZNRF3/RNF43 transmembrane ubiquitin ligase inhibition by the Wnt agonist R-spondin. *Nat. Commun.* 4:2787 doi: 10.1038/ncomms3787 (2013).

## Supplementary Material

Supplementary Information Supplementary Figures S1-S8, Supplementary Tables S1-S5 and Supplementary Reference

## Figures and Tables

**Table 1:** Data collection and refinement statistics.

**ZNRF3**_**ecto**_**/RNF43**_**ecto**_	**zZNRF3**	**mZNRF3**	**xZNRF3**	**—**	**—**	**mZNRF3**	**mZNRF3**	**xZNRF3**	**xRNF43**
**Rspo**_**Fu1–Fu2**_	**—**	**—**	**—**	**xRSPO2**	**xRSPO2–Pt**	**mRSPO2**	**xRSPO2**	**xRSPO2**	**xRSPO2**
Data collection									
Space group	P2_1_	P2_1_	P2_1_	P4_1_2_1_2	P4_1_2_1_2	P2_1_2_1_2_1_	P1	P2_1_	C2
Cell dimensions									
*a*, *b*, *c* (Å)	36.0, 53.3, 72.5	47.3, 57.8, 50.5	49.2, 58.7, 52.7	97.1, 97.1, 292.9	96.6, 96.6, 290.1	59.8, 77.2, 130.6	36.4, 71.0, 72.0	56.0, 81.2, 71.6	88.9, 35.8, 87.9
*α*, *β*, *γ* (°)	90, 102.1, 90	90, 97.6, 90	90, 93.6, 90	90, 90, 90	90, 90, 90	90, 90, 90	109.2, 101.7, 101.3	90, 113.0, 90	90, 114.6, 90
Resolution (Å)*	42.59–1.60 (1.63–1.60)	37.84–2.00 (2.05–2.00)	37.66–2.40 (2.49–2.40)	39.69–2.20 (2.25–2.20)	39.46–3.20 (3.46–3.20)	66.46–2.80 (2.97–2.80)	38.89–2.40 (2.49–2.40)	65.94–2.10 (2.16–2.10)	32.76–2.70 (2.83–2.70)
*R*_merge_	0.074 (0.359)	0.089 (0.353)	0.067 (0.478)	0.106 (0.852)	0.218 (1.443)	0.116 (1.345)	0.073 (0.605)	0.115 (0.788)	0.147 (0.550)
*I*/*σI*	16.1 (2.3)	9.7 (1.9)	8.7 (1.8)	10.3 (2.0)	23.2 (2.5)	16.0 (2.3)	15.0 (2.6)	17.1 (2.6)	9.5 (2.2)
Completeness (%)	99.8 (99.0)	91.8 (78.6)	99.2 (99.3)	99.1 (99.9)	99.9 (99.9)	99.9 (100)	78.5 (29.2)	93.7 (56.5)	95.8 (97.8)
Redundancy	11.0 (10.9)	3.6 (2.4)	3.0 (3.1)	6.8 (6.6)	50.6 (15.1)	14.4 (14.6)	6.6 (5.8)	17.0 (7.8)	3.8 (3.0)
									
Refinement									
Resolution (Å)*	42.59–1.60 (1.63–1.60)	37.84–2.00 (2.05–2.00)	37.66–2.40 (2.49–2.40)	39.69–2.20 (2.25–2.20)		66.46–2.80 (2.97–2.80)	38.89–2.40 (2.49–2.40)	65.94–2.10 (2.16–2.10)	32.76–2.70 (2.83–2.70)
No. of reflections	34,163	15,439	12,423	67,674		14,706	18,529	31,015	6,517
*R*_work_/*R*_free_	0.222/0.258	0.200/0.276	0.224/0.299	0.223/0.270		0.236/0.323	0.195/0.273	0.188/0.246	0.317/0.395
No. of atoms									
Protein	2,121	2,327	2,220	6,807		3,967	3,815	4,145	1,679
Water	79	29	9	292		—	—	220	—
Ligands	—	—	—	—		1	—	—	—
*B*-factors (Å^2^)									
Protein	41.5	48.6	64.7	47.9		86.2	79.8	35.4	60.4
Water	37.8	42.7	51.0	40.6		—	—	36.8	—
Ligands	—	—	—	—		79.1	—	—	—
*r.m.s.d.*									
Bond lengths (Å)	0.008	0.013	0.012	0.012		0.013	0.015	0.013	0.004
Bond angles (°)	1.199	1.591	1.631	1.418		1.656	1.864	1.604	0.748
Number of monomers or 1:1 complexes	2	3	2	8		2	2	2	1
Dimeric architecture	No	Yes	Yes	—		Yes	Yes	Yes	No
Protein Data Bank code	4C84	4C86	4C8T	4C8V		4C99	4C9A	4C9R	4C9V

^*^Highest resolution shell is shown in parenthesis. Statistics of additional structures can be found in Supplementary Table S1.
