# Supplementary material for: Structural and molecular basis of ZNRF3/RNF43 transmembrane ubiquitin ligase inhibition by the Wnt agonist R-spondin
Source: Nat Commun. 2013 Nov 14;4:2787. doi: 10.1038/ncomms3787 (PMC3905715; doi:10.1038/ncomms3787)
Supplement: Supplementary Information — Supplementary Figures S1-S8, Supplementary Tables S1-S5 and Supplementary Reference [file ncomms3787-s1.pdf]

Supplementary Figures

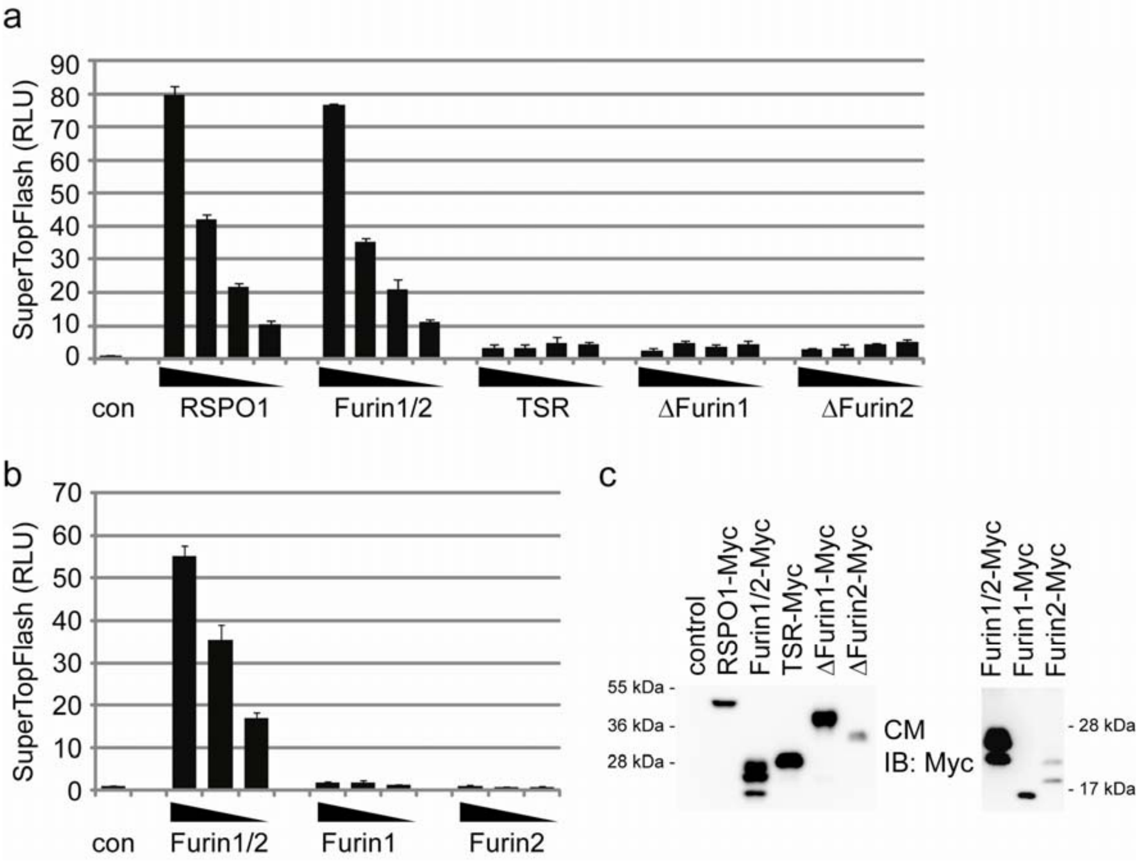

Supplementary Figure S1: RSPO1 Furin domains 1 and 2 are required for Wnt pathway activation assayed by the SuperTopFlash reporter. (a) Decreasing doses (100ng, 25ng, 6.25ng, 1.6ng) co-transfected for Human RSPO1-Myc and deletion constructs TSR-Myc, ΔFurin 1-Myc and ΔFurin2-Myc. Due to lower expression levels, twice as much Furin1/2-Myc (200ng, 50ng, 12.5ng, 3.1ng) was transfected. RLU (relative luciferase units). Error bars represent standard deviations from three replicates. **(b)** Individual Furin domains (expressed at 50ng, 12.5ng, 3.1ng) are not sufficient to activate the Wnt reporter. **(c)** Western blot showing amounts of secreted RSPO1-Myc proteins detected in the conditioned media (CM).

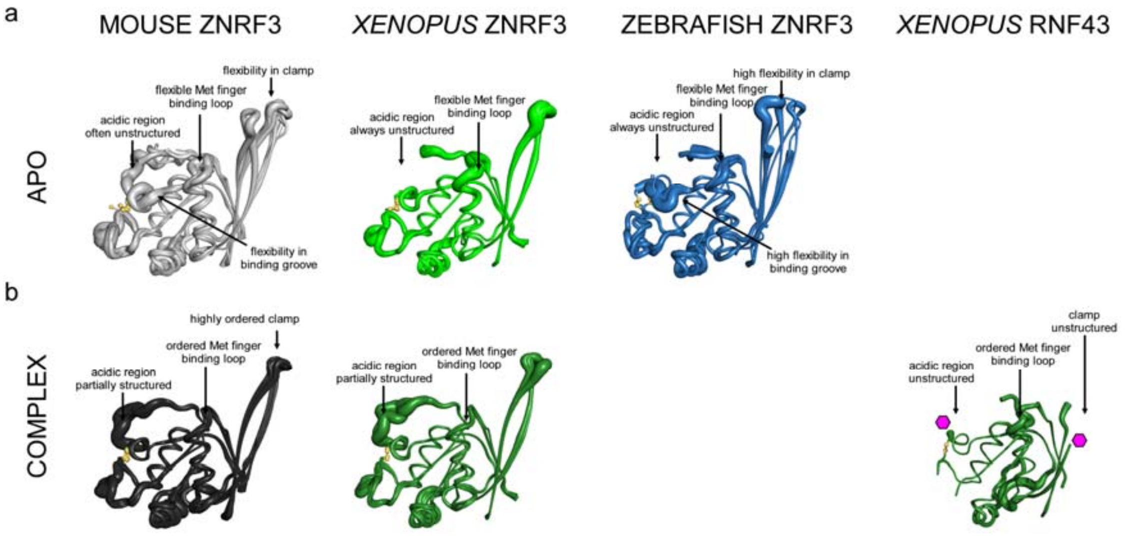

Supplementary Figure S2: Conserved fold and flexibility in apo ZNRF3$_{ecto}$ structures (a) vs. induced structure in ligand-bound structures (b). All crystallographic models of the ZNRF3 ectodomain of apo vs. complex structures of a single species have been superimposed and displayed using the "putty" option of Pymol. The B factor of Cα atoms is displayed by the diameter of the Cα tube. Hence, well ordered Cα atoms appear small and those with a high coordinate variation are smeared out. Overall the fold is preserved across species and no large structural changes occur upon ligand binding. Apo structures display three regions with high coordinate variation: the long β1-β2 hairpin, the αC-β7 loop and the elaborate β3-β4 loop that forms part of the binding groove and includes an acidic region with the sequence ^{105}NNNDEEDLYE^{114} (mouse numbering). In most apo structures this acidic region is not defined at all. Complex structures show far less coordinate variation in these regions. For the structure of xRNF43 two glycosylation sites are indicated by a pink hexagon.

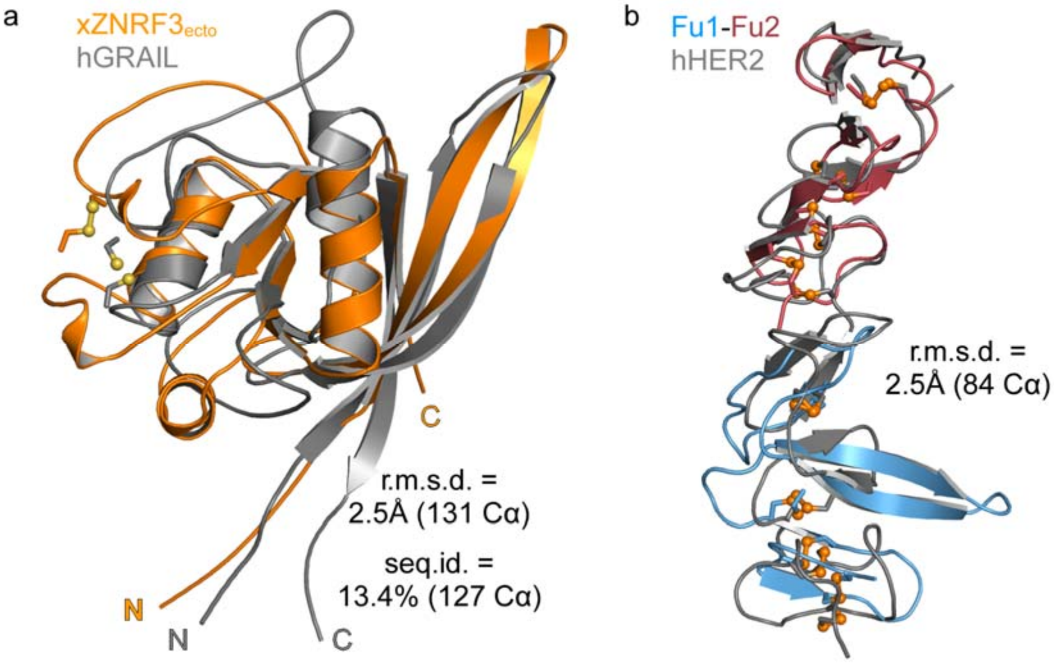

Supplementary Figure S3: Structural relatives of ZNRF3$_{ecto}$ and RSPO2$_{Fu1\text{-}Fu2}$. (a) Preserved fold of the ectodomains of membrane-tethered E3 ubiquitin ligases ZNRF3 and GRAIL. Structures of xZNRF3$_{ecto}$ (orange) and the deposited GRAIL ectodomain structure (pdb accession 3ICU in gray) have been superimposed using the SSM algorithm. **(b)** The closest structural relatives of Rspo proteins are the Furin-like cysteine rich domains of ErbB2. The Fu1 and 2 repeats of xRspo2 have been superimposed separately onto the structure of HER2 (pdb code 1N8Z, residues 488-603). The combined r.m.s.d. is indicated.

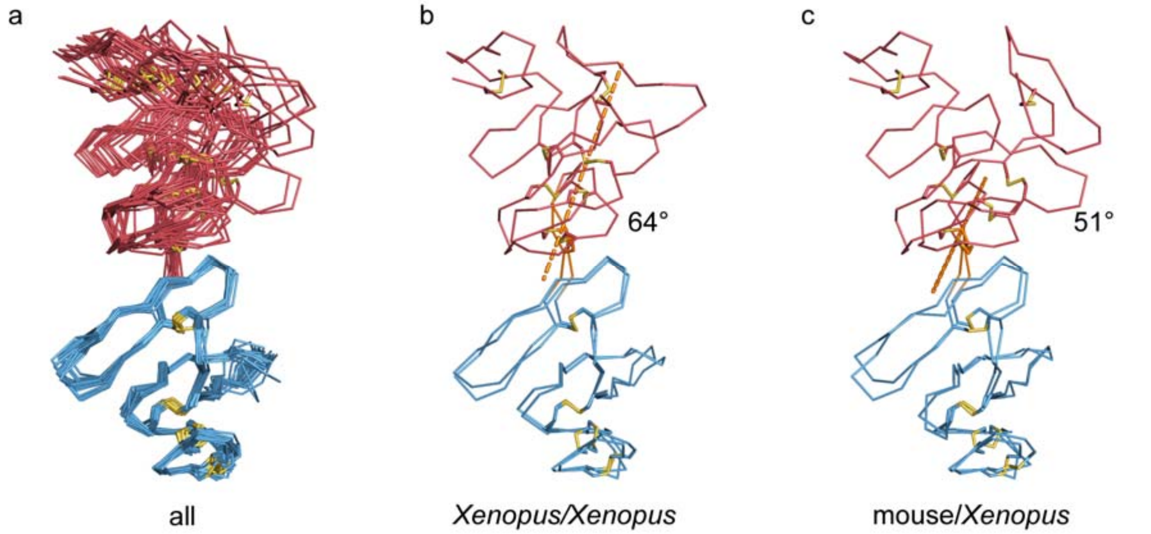

Supplementary Figure S4: High relative rotational flexibility of the Furin-like repeats of Rspos. (a), 22 Receptor bound and free $Rspo_{Fu1-Fu2}$ crystal structures have been superimposed based on Fu1. We see no correlation between relative domain orientation and receptor binding. **(b)**, the two xRspo2 $_{Fu1-Fu2}$ structures differing most in their relative orientation of Fu1 are shown. Their respective Fu1 domains are related by a 64° rotation around the axis shown in orange. **(c)**, the two most different xRspo2 $_{Fu1-Fu2}$ and mRspo2 $_{Fu1-Fu2}$ are shown. They differ by a domain rotation around the axis in orange. In both cases the program DYNDOM[49] identifies the regions between the two repeats as hinge.

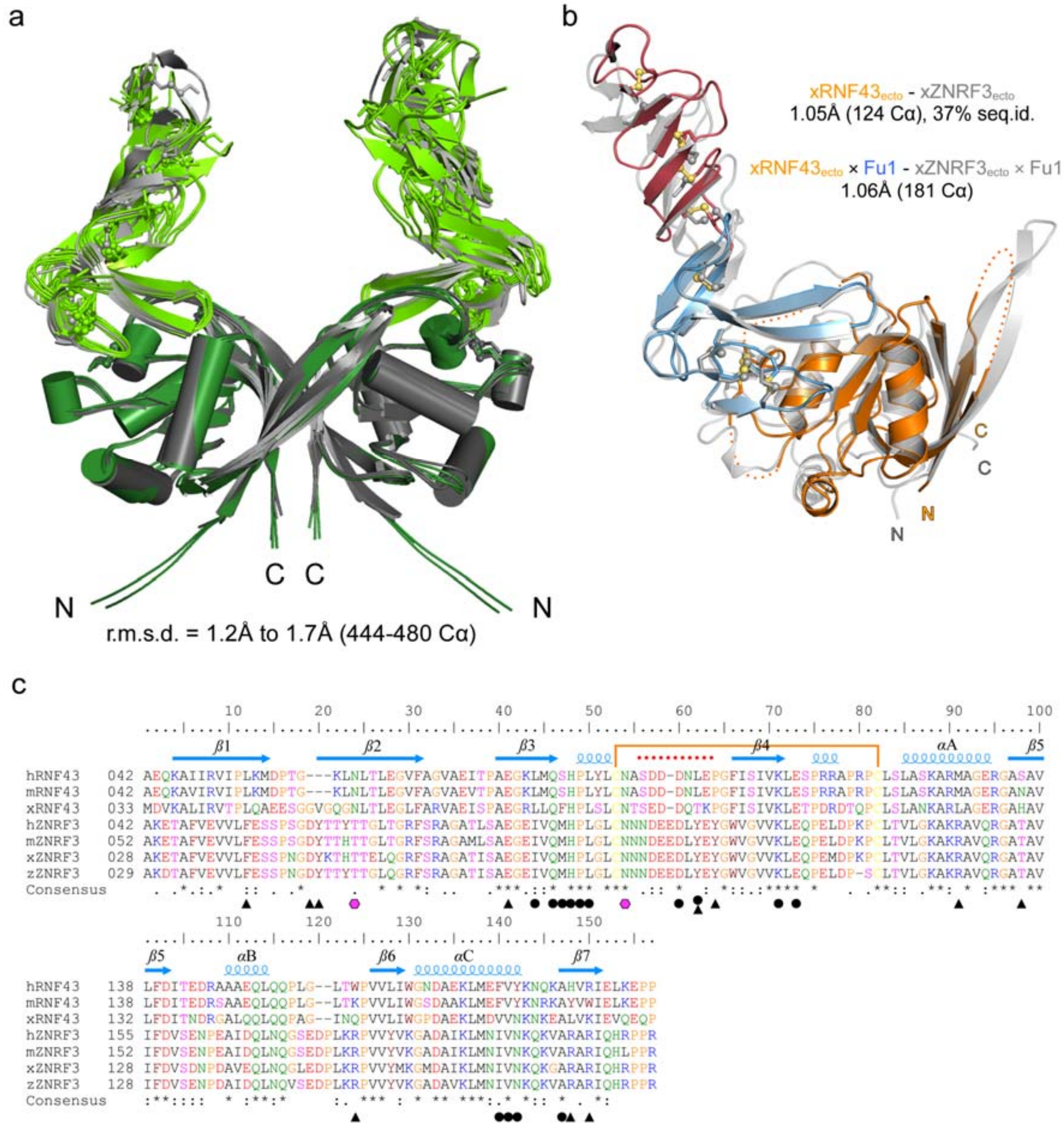

Supplementary Figure S5: Comparison of $Rspo2_{Fu1\text{-}Fu2}$ complexes of $ZNRF3_{ecto}$ and $RNF43_{ecto}$. (a) Consistent crystallization of ZNRF3ecto-RspoFu1-Fu2 as a 2:2 complex. Seven independent structures have been superimposed by the SSM algorithm using all atoms. Mouse proteins are in gray and *Xenopus* proteins in green. The figure highlights a consistent dimerization and a strong conservation of the dimeric receptor structure. Flexibility is seen in the Fu2 repeat of the ligand, which is not involved in ZNRF3 binding. **(b)** 1:1 complex of $xRNF43_{ecto}$ and $xRSPO2_{Fu1\text{-}Fu2}$. Shown is a superposition with the corresponding ZNRF3 complex in white. The statistics of the two superpositions highlight the fact that Fu1 engages the exact same interface on both receptors in identical relative orientation. As in (a), Fu2 engages in a different orientation due to not being involved in receptor binding. A comparison of our RNF43 structure with that reported in the recently published LGR5-RSPO1-RNF43 complex structure (pdb id 4KNG[25]) suggests that the previous structure of RNF43 is in error in the C-terminal region K178-P192 being out of register by two residues. **(c)** Sequence alignment between RNF43 and ZNRF3 of several species (ectodomain only).

Residues involved in ligand binding are highlighted with a circle and those involved in ZNRF3 dimerization with a triangle. The two N-glycosylation sites of RNF43 are highlighted with a pink hexagon and the acidic region discussed in the main text is marked by a red dotted line.

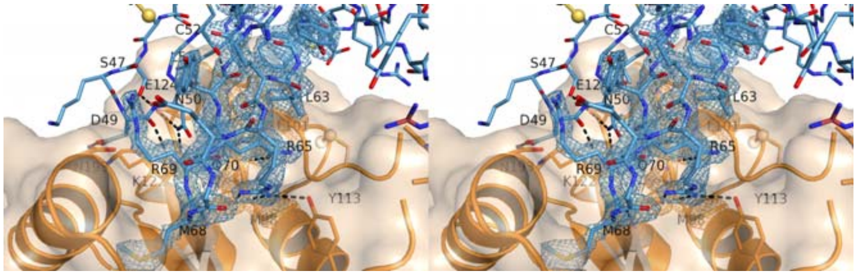

Supplementary Figure S6: The xZNRF3$_{\mathbf{ecto}}$-xRspo2$_{\mathbf{Fu1\text{-}Fu2}}$ interface. The stereo image is related to Fig. 1g and shows the final weighted 2Fo-Fc electron density map around residues Q97-L101 (ZNRF3) and F61-E73 (RSPO2) contoured at 2σ.

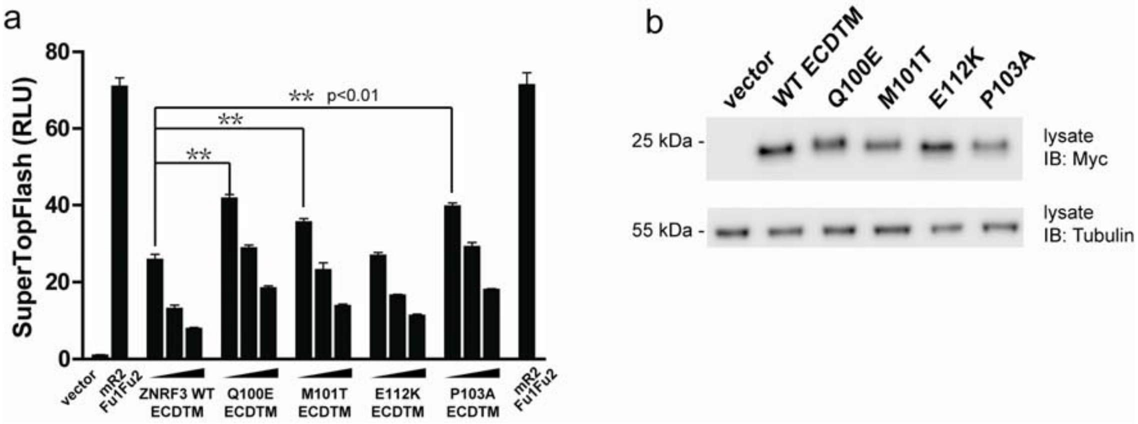

Supplementary Figure S7: ZNRF3 mutants that are defective in RSPO binding display reduced dominant negative activity in the context of ZNRF3 ECDTM. (a) Mouse Rspo2 Fu1Fu2-Myc transfected at 15 ng activates the SuperTopFlash reporter. Co-transfection of Rspo2 Fu1Fu2-Myc with increasing doses (0.4ng, 2ng, 10ng) of ZNRF3 ECDTM decreases Wnt reporter activity as reported[16], likely via titrating Rspo. The P103A ECDTM previously shown to be defective in Rspo binding is less effective in this dominant negative activity than ZNRF3 ECDTM. Human ZNRF3 mutants Q100E, M101T and E112K (analogous to mouse Znrf3 Q97E, M98T and E109K mutants used in Figure 4) were compared to ZNRF3 ECDTM and P103A ECDTM. The Q100E and M101T mutants showed significant reductions in dominant negative activity (Student's T test comparing WT 0.4ng dose to mutant), consistent with the loss of Rspo interaction detected by SPR. Error bars represent standard deviations from three replicates. **(b)** Western blots showing similar expression levels of the WT and mutant ZNRF3 ECDTM proteins (Myc-tagged) from whole cell lysates.

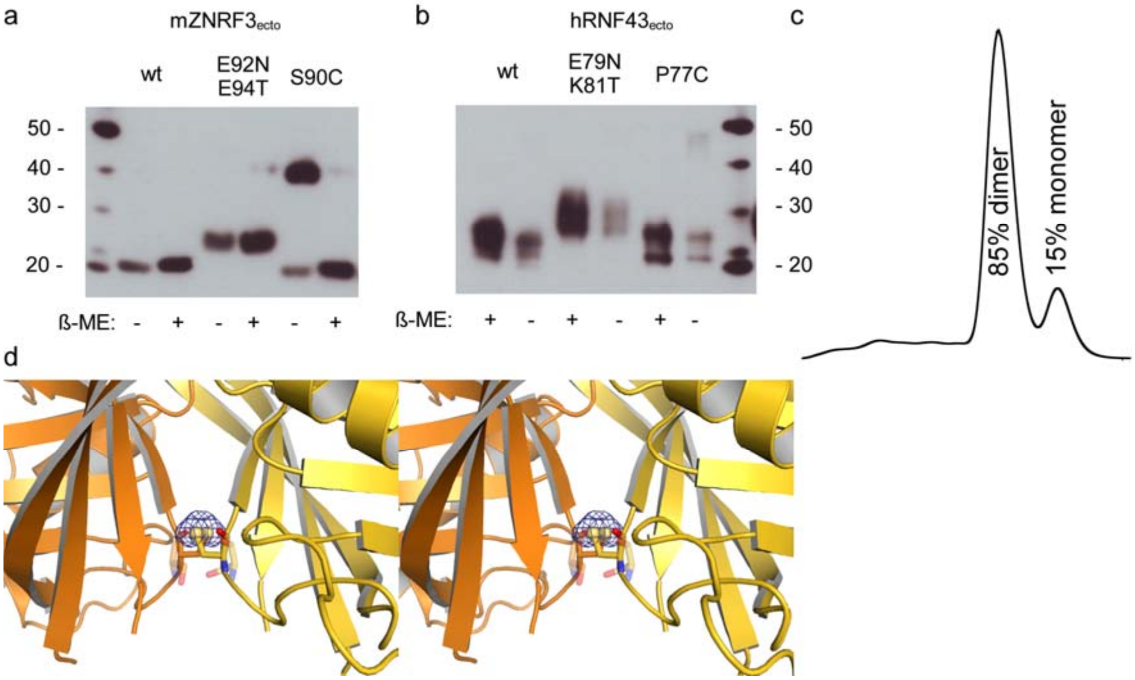

Supplementary Figure S8: Dimer interface mutants of mZNRF3$_{ecto}$ and hRNF43$_{ecto}$. (a) and **(b)** the incorporation of a potential N-glycosylation site in the dimer interface of ZNRF3 and the corresponding region in RNF43$_{ecto}$ leads to a slower migration in SDS PAGE suggesting full glycosylation in both proteins. Incorporation of a cysteine close to the symmetry axis leads to almost quantitative spontaneous cross linking in ZNRF3 but not in RNF43. **(c)** gel filtration elution profile of mZNRF3$_{ecto}$ S90C. A crystal structure of mZNRF3$_{ecto}$ S90C **(d)** shows that disulfide formation requires only subtle backbone distortion.

Supplementary Table S1

Data collection and refinement statistics for additional apo and complex $ZNRF3_{ecto}$ and $Rspo2_{Fu1\text{-}Fu2}$ structures.

$ZNRF3_{ecto}$	zZNRF3	mZNRF3	mZNRF3	mZNRF3	mZNRF3 S90C	xZNRF3	-	mZNRF3	xZNRF3
$Rspo_{Fu1\text{-}Fu2}$	-	-	-	-	-	-	xRSPO2	xRSPO2	xRSPO2
Crystal form	II	II	III	IV	V	II	II	II	II
**Data collection**									
Space group	$P4_12_12$	C2	$P2_12_12_1$	C2	$P2_12_12$	$P2_12_12_1$	P422	P1	$P222_1$
cell dimension									
a, b, c (Å)	52.4, 52.4, 204.2	71.3, 76.5, 93.3	58.2, 69.6, 75.4	109.2, 112.6, 62.2	58.7, 74.2, 42.8	58.3, 67.3, 75.5	87.1, 87.1, 77.0	71.0, 71.3, 72.1	57.6, 208.9, 49.8
α, β, γ (°)	90, 90, 90	90, 93.3, 90	90, 90, 90	90, 91.0, 90	90, 90, 90	90, 90, 90	90, 90, 90	69.3, 61.5, 84.3	90, 90, 90
Resolution (Å)	46.60-2.50 (2.61-2.50)	52.09-2.70 (2.83-2.70)	46.08-2.40 (2.50-2.40)	49.03-2.69 (2.82-2.69)	46.07-2.10 (2.17-2.10)	75.68-3.01 (3.21-3.01)	87.12-3.10 (3.31-3.10)	35.34-3.00 (3.24-3.00)	52.23-3.00 (3.18-3.00)
R_{merge}	0.088 (0.531)	0.056 (1.037)	0.068 (0.578)	0.133 (0.025)	0.071 (0.632)	0.368 (1.357)	0.186 (0.758)	0.073 (0.297)	0.138 (0.818)
$I/\sigma I$	10.1 (2.3)	20.6 (2.1)	14.4 (1.9)	7.8 (3.1)	11.5 (2.0)	8.0 (2.9)	6.8 (2.4)	6.4 (1.8)	7.2 (1.9)
Completeness (%)	98.0 (98.9)	99.5 (98.9)	87.3 (54.3)	90.4 (56.9)	99.8 (99.8)	99.6 (99.5)	99.1 (99.9)	98.6 (98.1)	93.9 (95.8)
Redundancy	4.2 (4.3)	9.1 (8.7)	7.1 (3.7)	4.5 (2.1)	4.9 (4.9)	19.9 (20.2)	4.8 (5.0)	2.0 (2.0)	3.7 (3.9)
**Refinement**									
Resolution (Å)	46.60-2.50 (2.61-2.50)	52.09-2.70 (2.83-2.70)	46.08-2.40 (2.50-2.40)	49.03-2.69 (2.82-2.69)	46.07-2.10 (2.17-2.10)	75.68-3.01 (3.21-3.01)	87.12-3.10 (3.31-3.10)	35.34-3.00 (3.24-3.00)	52.23-3.00 (3.18-3.00)
No. reflections	9792	12423	10424	17441	9382	5981	5423	21538	12862
R_{work} / R_{free}	0.221 / 0.301	0.224 / 0.299	0.236 / 0.320	0.229 / 0.338	0.204 / 0.286	0.281 / 0.351	0.230 / 0.313	0.245 / 0.334	0.227 / 0.310
No. atoms									
Protein	2123	3129	2156	4174	1108	2206	1549	7644	3856
Water	3	-	-	-	-	-	-	-	-
Ligands	-	-	2	-	21	1	-	-	-
B-factors (Å^2)									
Protein	47.1	65.8	63.2	69.0	59.3	67.3	59.5	100.3	68.8

Water	34.3	-	-	-	57.9	-	-	-	-
Ligands	-	-	34.6	-	-	53.4	-	-	-
R.m.s.deviations									
Bond lengths (Å)	0.011	0.012	0.014	0.013	0.015	0.011	0.010	0.010	0.013
Bond angles (°)	1.464	1.518	1.706	1.687	1.864	1.476	1.369	1.509	1.648
number of monomers or 1:1 complexes	2	2	2	4	1	2	2	4	2
dimeric architecture	yes	yes	yes	no	yes	yes		yes	yes
PDB code	4C85	4C8A	4C8C	4C8F	4C8P	4C8U	4C8W	4C9E	4C9U

Values in brackets refer to the highest resolution shell.

Supplementary Table S2

Interface areas calculated from crystal structures using PISA.

**Crystal form**	**Dimer interface** interacting chains interaction area (Å^2)	**Rspo interface** interacting chains interaction area (Å^2)
zZNRF3_apo_I*	A/B 743	
zZNRF3_apo_II	A/B 768	
mZNRF3_apo_I	A/B 955	
mZNRF3_apo_II	A/B 1004<br>C/C' 1002	
mZNRF3_apo_III	A/B 1065	
mZNRF3_apo_IV*	B/C 1099<br>A/D 1057	
xZNRF3_apo_I	A/B 1087	
xZNRF3_apo_II	A/B 1064	
**apo average +/- SD**	**992 +/- 109**	
mZNRF3_mRspo2	A/C 1170	
mZNRF3_xRspo2_I	A/C 1184	A/B 907<br>C/D 1024
mZNRF3_xRspo2_II	A/C 1228<br>E/G 1178	A/B 894<br>C/D 1000<br>E/F 1094<br>G/H 1022
xZNRF3_xRspo2_I	A/C 1203	A/B 1021<br>C/D 1070
xZNRF3_xRspo2_II	A/A' 1182<br>C/C' 1099	A/B 1043<br>C/D 1007
**ZNRF3 complexes average +/- SD**	**1178 +/- 40**	**1014 +/- 63**
xRNF43_xRspo2*	A/A' 334	A/B 703
**all complexes average +/- SD**	**-**	**990 +/- 105**

*These crystal forms show no parallel orientation of the protomers. The largest identified crystal interface areas have thus not been included in the calculation of average interface area values.

Supplementary Table S3

Pairwise superposition of $mZNRF3_{ecto}$ dimer of apo and complex structures. Shown are r.m.s.d. values and the number of aligned Cα atoms in brackets. Comparisons between apo/apo and complex/complex structures are shown above the diagonal whereas those comparing apo to complex structures are shown below.

	mZNRF3_apo_I_AB	mZNRF3_apo_II_AB	mZNRF3_apo_II_CC'	mZNRF3_apo_III_AB	mZNRF3_mRspo2_AC	mZNRF3_xRspo2_I_AC	mZNRF3_xRspo2_I_EG	mZNRF3_xRspo2_II_AC
mZNRF3_apo_I_AB	-	0.85 (281)	0.98 (286)	0.66 (278)				
mZNRF3_apo_II_AB		-	0.85 (293)	0.72 (287)				
mZNRF3_apo_II_CC'			-	0.78 (287)				
mZNRF3_apo_III_AB				-				
mZNRF3_mRspo2_AC	0.96 (286)	0.72 (292)	0.93 (304)	0.63 (286)	-	0.60 (303)	0.63 (303)	0.61 (303)
mZNRF3_xRspo2_I_AC	1.01 (291)	0.79 (292)	0.89 (302)	0.62 (288)	0.58 (303)	-	0.67 (305)	0.47 (305)
mZNRF3_xRspo2_I_EG	1.11 (290)	0.93 (291)	0.96 (301)	0.79 (289)	0.63 (303)		-	0.55 (305)
mZNRF3_xRspo2_II_AC	0.88 (289)	0.71 (289)	0.87 (302)	0.58 (288)	0.61 (303)			-

Supplementary Table S4

Pairwise superposition of $xZNRF3_{ecto}$ dimer of apo and complex structures. Shown are r.m.s.d. values and the number of aligned Cα atoms in brackets. Comparisons between apo/apo and complex/complex structures are shown above the diagonal whereas those comparing apo to complex structures are shown below.

	xZNRF3_apo_I_AB	xZNRF3_apo_II_AB	xZNRF3_xRspo2_I_AA'	xZNRF3_xRspo2_I_CC'	xZNRF3_xRspo2_II_AC
xZNRF3_apo_I_AB	-	0.61 (290)			
xZNRF3_apo_II_AB		-			
xZNRF3_xRspo2_I_AA'	0.81 (289)	0.79 (292)	-	0.56 (308)	0.69 (319)
xZNRF3_xRspo2_I_CC'	0.79 (290)	0.78 (292)		-	0.70 (307)
xZNRF3_xRspo2_II_AC	0.69 (290)	0.62 (292)			-

Supplementary Table S5

Protein sequence identities between ZNRF3 and RNF43 ectodomains from several species. Comparisons within one receptor group are shown above the diagonal whereas those comparing ZNRF3 to RNF43 are shown below.

	hZNRF3	mZNRF3	xZNRF3	zZNRF3	hRNF43	mRNF43	xRNF43
hZNRF3	1.00	0.98	0.90	0.94			
mZNRF3		1.00	0.90	0.92			
xZNRF3			1.00	0.90			
zZNRF3				1.00			
hRNF43	0.39	0.39	0.39	0.39	1.00	0.93	0.65
mRNF43	0.38	0.39	0.39	0.38		1.00	0.64
xRNF43	0.38	0.38	0.37	0.37			1.00

Supplementary References

49. Hayward, S. & Berendsen, H. J. Systematic analysis of domain motions in proteins from conformational change: new results on citrate synthase and T4 lysozyme. *Proteins* **30**, 144-154 (1998).
